# Prediction of EGFR and KRAS mutation in non-small cell lung cancer using quantitative ^18^F FDG-PET/CT metrics

**DOI:** 10.18632/oncotarget.17782

**Published:** 2017-05-10

**Authors:** Ryogo Minamimoto, Mehran Jamali, Olivier Gevaert, Sebastian Echegaray, Amanda Khuong, Chuong D. Hoang, Joseph B. Shrager, Sylvia K. Plevritis, Daniel L. Rubin, Ann N. Leung, Sandy Napel, Andrew Quon

**Affiliations:** ^1^ Department of Radiology, Stanford University, Stanford, CA, USA; ^2^ Department of Cardiothoracic Surgery, Stanford University, Stanford, CA, USA

**Keywords:** ^18^FDG-PET/CT, heterogeneity, KRAS gene mutation, EGFR gene mutation, NSCLC

## Abstract

This study investigated the relationship between epidermal growth factor receptor (*EGFR*) and Kirsten rat sarcoma viral oncogene homolog (*KRAS*) mutations in non-small-cell lung cancer (NSCLC) and quantitative FDG-PET/CT parameters including tumor heterogeneity. 131 patients with NSCLC underwent staging FDG-PET/CT followed by tumor resection and histopathological analysis that included testing for the *EGFR* and *KRAS* gene mutations. Patient and lesion characteristics, including smoking habits and FDG uptake parameters, were correlated to each gene mutation. Never-smoker (*P* < 0.001) or low pack-year smoking history (*p* = 0.002) and female gender (*p* = 0.047) were predictive factors for the presence of the EGFR mutations. Being a current or former smoker was a predictive factor for the KRAS mutations (*p* = 0.018). The maximum standardized uptake value (SUV_max_) of FDG uptake in lung lesions was a predictive factor of the *EGFR* mutations (*p* = 0.029), while metabolic tumor volume and total lesion glycolysis were not predictive. Amongst several tumor heterogeneity metrics included in our analysis, inverse coefficient of variation (1/COV) was a predictive factor (*p* < 0.02) of *EGFR* mutations status, independent of metabolic tumor diameter. Multivariate analysis showed that being a never-smoker was the most significant factor (*p* < 0.001) for the *EGFR* mutations in lung cancer overall. The tumor heterogeneity metric 1/COV and SUV_max_ were both predictive for the *EGFR* mutations in NSCLC in a univariate analysis. Overall, smoking status was the most significant factor for the presence of the *EGFR* and *KRAS* mutations in lung cancer.

## INTRODUCTION

Epidermal growth factor receptor (*EGFR*) [1, 2], Kirsten rat sarcoma viral oncogene homolog (*KRAS*) [3] and anaplastic lymphoma kinase (*ALK*) [4] are all significant biomarkers for the management of non-small-cell lung cancer (NSCLC). *EGFR* is a member of a larger family of closely related transmembrane receptor tyrosine kinases (TK), which activate cell growth and replication, differentiation and survival [5, 6]. Mutations in the TK domain of the *EGFR* in NSCLC predict the response to TK inhibitors such as Gefitinib and Erlotinib [7–9]. *KRAS* exists downstream of *EGFR* and the *EGFR* pathway is altered by *KRAS* mutation [3]. However *KRAS* mutations are associated with lack of activity of the TK inhibitors [10]. *ALK*, the downstream serine-threonine kinase of *EGFR* signaling, rearranged tumors are not sensitive to *EGFR* TK inhibitors, but they are sensitive to *ALK* specific TK inhibitors such as Crizotinib [4]. Akt signaling is one of the main *EGFR* signaling pathways and includes the upregulation of glucose transporter (GLUT) 1 and 4 transporters [11, 12]. As a result, Akt activation may have a close relationship with *EGFR* mutations and fluorodeoxyglucose (FDG) uptake in NSCLC. [13, 14]. While the relationship between FDG uptake and *EGFR* mutations in NSCLC has previously been noted to have contradictory results [15, 16], and one notable study has shown that the *KRAS* mutations in lung cancer showed significantly higher FDG uptake than wild type (WT) cancer [17]. Tumor heterogeneity relates to both tumor development and therapeutic outcomes [18]. Moreover, clonal heterogeneity can be identified within the primary tumor ahead of identification of the metastases [19]. Intra-tumor heterogeneity appears to correlate to the *EGFR* mutations in NSCLC and may predict tumor responsiveness to TK inhibitors therapy [20, 21].

FDG uptake usually is not homogeneously distributed within the tumor, which can be caused by variations in necrosis [22], cellular proliferation [23] and hypoxia [24]. High intratumor heterogeneity therefore could potentially serve as a prognostic factor in NSCLC [25].

In this study, we investigate if there exists a relationship between *EGFR* mutations and/or *KRAS* mutations in NSCLC status and several FDG-PET/CT parameters such as maximum standardized uptake value (SUV_max_), metabolic tumor volume (MTV), total lesion glycolysis (TLG), and tumor heterogeneity, in order to determine the FDG-PET/CT metrics that are most predictive of a gene mutation. Subjects were recruited and enrolled in this trial if they had suspected NSCLC based on a diagnostic CT scan. Subsequently, patients underwent a battery of testing that included FDG-PET/CT scanning and gene mutation testing. Gene mutations were investigated using tissues from surgically resected tumor for all patients.

## RESULTS

### Patient characteristics

The total number of enrolled patients was 182. Fifty-one cases were excluded due to any one or more of the following reasons: 1) margin of lesion not well defined (pneumonic form and central obstructive lesions on preoperative CT which was confirmed by a board-certified radiologist specializing in thoracic imaging), 2) gene mutation analysis not performed, and 3) histologic subtypes other than adenocarcinoma by pathological diagnosis. After the above exclusions, 131 patients (male: 86, female: 45, mean age ± SD: 67 ± 10, range 24–81yrs) met the eligibility criteria for this study and the clinical characteristics of these patients are listed in Table 1. In this study, lung cancer was more frequently identified in males (86/131, 65.6%), but there was no significant age difference between males and females. Lung carcinoma was primarily found in patients classified as current and former smokers (75.8%). The characteristics of identified lung lesions are shown in Table 2. The majority of patients enrolled in this study was clinical stage of IA or IB disease (92/131, 70.2%).

**Table 1:** Patient and lesion characteristics

Characteristic	Number	Percentage
Gender		
Male	86	65.6
Female	45	34.4
Total	131	100
Mean Age (range)		
Male	68 ± 10 (24–86)	-
Female	67 ± 10 (45–81)	-
Total	67 ± 10 (24–86)	-
Smoking status		
Current/former smoker	99 (22/77)	75.8
Never smoker	32	24.2
Location		
Right lobe (RUL/RML/RLL)	81 (48/11/22)	61.8
Left lobe (LUL/LLL)	50 (32/18)	38.2
Pathology		
Adenocarcinoma	131	100.0
Clinical and pathological staging		
IA	66	50.4
IB	26	19.8
IIA	12	9.2
IIB	10	7.6
IIIA	12	9.2
IV	2	1.5
Undefined	3	2.3
Gene mutation (positive/negative/N/A)		
EGFR	32/95/4	-
KRAS	31/95/5	-

RUL: right upper lobe, RML: right middle lobe, RLL: right lower lobe, LUL: left upper lobe, LLL: left lower lobe

**Table 2:** FDG uptake at the normal lung field (*n* = 131)

Area of lung	RUF	LUF	RMF	LMF	RLF	LLF	Blood pool
SUVmean	0.5 ± 0.1	0.5 ± 0.1	0.4 ± 0.2	0.5 ± 0.2	0.6 ± 0.3	0.6 ± 0.2	1.7 ± 0.4
Range	0.2–0.9	0.2–1.1	0.1–1.3	0.2–1.2	0.1–1.8	0.3–1.3	0.8–2.9

RUF: right upper field, RMF: right middle field, RLF: right lower field, LUF: left upper field, LMF: left middle field, LLF: left lower field,

### Background FDG uptake in normal lung parenchyma

Results are shown in Table 3. No significant difference was found between left and right lobe for upper area, middle area and the lower area respectively. FDG uptake in lower area was higher than upper area (*p* < 0.001) and middle area (*p* < 0.001). No significant factor (age, sex, smoking status, pack years and gene mutation) could be identified for the FDG uptake for normal lung.

**Table 3:** Result in the FDG parameters

FDG-PET Parameter	Mean ± SD All	EGFR mutations		*P* value	KRAS mutations		*P* value
		(+)	(−)		(+)	(−)	
Metabolic tumor diameter (mm)	33 ± 27 (8–230)	27 ± 13 (8–53)	34 ± 30 (8–230)	0.60	36 ± 29 (8–135)	32 ± 27 (8–230)	0.75
SUVmax	6.3 ± 5.9 (0.7–36.7)	4.2 ± 3.8 (0.7–14.2)	6.9 ± 3.8 (0.8–36.7)	0.009	7.4 ± 7.6 (0.9–36.7)	5.9 ± 5.3 (0.7–29.6)	0.38
SUVmean	3.8 ± 2.7 (0.7–18.9)	3.1 ± 2.3 (0.8–10.0)	4.0 ± 2.9 (0.7–18.9)	0.09	4.2 ± 3.6 (0.7–18.9)	6.9 ± 3.8 (0.8–36.7)	0.67
TLG	109.2 ± 530.8 (0.4–5577.5)	17.6 ± 34.7 (0.4–162.3)	143.4 ± 623.5 (0.5–5577.5)	0.04	269.4 ± 1028.1 (0.7–5577.5)	61.4 ± 203.9 (0.4–1725.8)	0.45
MTV	14.5 ± 38.8 (0.3–295.1)	6.2 ± 10.4 (0.3–50.8)	17.6 ± 45.0 (0.3–295.1)	0.29	24.9 ± 65.8 (0.7–295.1)	11.2 ± 25.2 (0.3–137.9)	0.59
SD (>1cm)	1.20 ± 1.16 (0.08–6.07)	0.90 ± 1.17 (0.11–5.87)	1.27 ± 1.17 (0.08–6.07)	0.02	1.30 ± 1.30 (0.08–6.07)	1.16 ± 1.13 (0.11–5.96)	0.65
1/COV (> 1cm)	4.24 ± 2.01 (1.51–17.91)	5.10 ± 1.89 (1.70–9.09)	4.13 ± 2.34 (1.51–17.91)	0.003	4.35 ± 2.49 (1.70–15.13)	4.34 ± 2.21 (1.51–17.91)	0.75
AUC (> 1cm)	0.61 ± 0.12 (0.28–0.86)	0.66 ± 0.12 (0.33–0.81)	0.60 ± 0.12 (0.28– 0.86)	0.02	0.61 ± 0.12 (0.28–0.78)	0.61 ± 0.13 (0.33–0.86)	0.96
SD (> 2cm)	1.35 ± 1.25 (0.09–6.07)	0.95 ± 1.23 (0.11–5.87)	1.52 ± 1.25 (0.09–6.07)	0.006	1.45 ± 1.37 (0.09–6.07)	1.33 ± 1.23 (0.11–5.96)	0.76
1/COV (> 2cm)	3.96 ± 1.53 (1.51–8.93)	4.93 ± 1.81 (0.33–8.93)	3.59 ± 1.25 (1.51–7.67)	0.001	3.85 ± 1.36 (1.69–7.67)	3.98 ± 1.61 (1.51–8.93)	0.98
AUC (> 2cm)	0.59 ± 0.12 (0.28–0.81)	0.66 ± 0.12 (0.33–0.81)	0.57 ± 0.12 (0.28– 0.78)	0.007	0.61 ± 0.12 (0.28– 0.78)	0.58 ± 0.13 (0.33– 0.81)	0.37
SD (> 3cm)	1.71 ± 1.39 (0.09–6.07)	1.39 ± 1.58 (0.09–6.07)	1.83 ± 1.58 (0.09–6.07)	0.11	1.92 ± 1.69 (0.09–6.07)	1.71 ± 1.33 (0.19–5.96)	0.72
1/COV (> 3cm)	3.45 ± 1.48 (1.50–8.92)	4.03 ± 1.73 (1.70–7.42)	3.10 ± 1.07 (1.51–7.67)	0.12	3.55 ± 1.55 (1.70–7.67)	3.25 ± 1.23 (1.51–7.42)	0.38
AUC (> 3cm)	0.53 ± 0.12 (0.28–0.78)	0.57 ± 0.12 (0.33– 0.78)	0.51 ± 0.11 (0.28– 0.78)	0.08	0.54 ± 0.14 (0.28–0.78)	0.52 ± 0.11 (0.33– 0.77)	0.68

SUV: Standardized uptake value, SUVmax: Maximum SUV, TLG: Total lesion glycolysis, MTV: Metabolic tumor volume. SD: standard deviation, COV: coefficient of variation, AUC: area under the curve of the cumulative SUV-volume histogram, range shown in parenthesis.

### Gene mutation analysis

*EGFR* gene mutations were confirmed in 32 of the 127 patients (25.2%). The *KRAS* gene mutation was confirmed in 31 of 126 patients (24.6%).

FDG-PET/CT parameters showing a significant difference between *EGFR* (+) and *EGFR*- WT case were SUV_max_, TLG, SD, 1/COV and AUC. In cases where the metabolic tumor diameter was greater than 3 cm, *EGFR* (+) and *EGFR* – WT had no significant correlation to the metabolic tumor diameter, tumor volume and the remaining tumor heterogeneity parameters. Further, no PET parameters appeared to correlate to the presence or absence of *KRAS* mutations (Table 4).

**Table 4:** Association between each indexes and *EGFR and KRAS* mutations status based on univariate analysis (*p*-values)

Index	EGFR mutations	KRAS mutations	Association
Age	0.70	0.79	-
Gender	0.047	0.06	Female with EGFR mutations
Cancer staging	0.54	0.68	-
Smoking status (Current / former smoker vs never-smoker)	< 0.001	0.018	Never-smoker with EGFR mutations, Current / former smoker with KRAS mutations
Pack Years	0.002	0.21	Low pack year smoking history (mostly never-smoker regarded as smoking history with 0 year) with EGFR mutations.
Maximum metabolic tumor diameter	0.26	0.66	-
SUVmax	0.029	0.20	Higher SUVmax with EGFR mutations
MTV	0.16	0.09	-
TLG	0.26	0.06	-
SD (> 1 cm)	0.16	0.94	-
1/COV (> 1 cm)	0.014	0.94	Higher 1/COV with EGFR mutations
AUC (> 1 cm)	0.036	0.88	Higher AUC with EGFR mutations
SD (> 2 cm)	0.07	0.70	-
1/COV (> 2 cm)	< 0.001	0.73	Higher 1/COV with EGFR mutations
AUC (> 3 cm)	0.012	0.45	-
SD (> 3 cm)	0.44	0.46	-
1/COV (> 3 cm)	0.008	0.98	Higher 1/COV with EGFR mutations
AUC (> 3 cm)	0.07	0.49	-

MTV: metabolic tumor volume, TLG: total lesion glycolysis, SD: standard deviation, COV: coefficient of variation, AUC: area under the curve of the cumulative SUV-volume Histogram. Measurements within parentheses are indicated maximum metabolic tumor diameter

The univariate analysis between several parameters and *EGFR* and *KRAS* mutation are shown in Table 5. Never-smoker (i.e. no prior smoking history), low-pack-year smoking history, and female gender were significant factors for *EGFR* mutation and smoker (current and former) was a significant factor for *KRAS* mutation. The SUV_max_ of FDG uptake in lung lesion was significant predictor, but those of MTV and TLG were not significant. Of the multiple parameters regarding tumor heterogeneity, 1/COV was the only parameter that was predictive of the *EGFR* mutation that was not effected or dependent on the metabolic tumor volume diameter. The multivariate analysis showed smoking status was most significant predictor for *EGFR* mutation in lung cancer. No parameters were identified that was predictive or significantly correlated to the *KRAS* mutation in lung cancer. The number of cases with each index evaluated in this study are shown in Table 6.

**Table 5:** Multivariate analysis for the association between each indexes and EGRF mutation status (*p*- values)

Index	EGFR mutations	Association
Gender	0.389	−
Smoking status (Current/former smoker vs never-smoker)	< 0.001	Never – smoker with EGFR mutations
SUVmax	0.378	−
1/COV (> 1 cm)	0.456	−

COV: coefficient of variation.

**Table 6:** Number of cases with each index

Index	EGFR mutations (+/−)	KRAS mutations (+/−)	Current/former smoker/never-smoker
Age	32/95	31/95	22/77/32
Gender male	16/66	25/59	17/56/13
Gender female	16/29	6/36	5/21/19
Smoking status Current	1/20	8/14	-
Former smoker	13/62	20/54	-
Never-smoker	18/13	3/27	-
Pack Years	32/95	31/95	22/77/32
Maximum metabolic tumor diameter	32/95	31/95	22/77/32
SUVmax	32/95	31/95	22/77/32
MTV	32/95	31/95	22/77/32
TLG	32/95	31/95	22/77/32
SD (> 1 cm)	27/85	28/84	20/70/26
1/COV (> 1 cm)	27/85	28/84	20/70/26
AUC (> 1 cm)	27/85	28/84	20/70/26
SD (> 2 cm)	24/65	24/64	13/59/21
1/COV (> 2 cm)	24/65	24/64	13/59/21
AUC (> 2 cm)	24/65	24/64	13/59/21
SD (> 3 cm)	12/42	13/40	5/37/14
1/COV (> 3 cm)	12/42	13/40	5/37/14
AUC (> 3 cm)	12/42	13/40	5/37/14

MTV: metabolic tumor volume, TLG: total lesion glycolysis, SD: standard deviation, COV: coefficient of variation, AUC: area under the curve of the cumulative SUV-volume Histogram. Measurements within parentheses are indicated maximum metabolic tumor diameter

## DISCUSSION

In the present study, we found that patients that were categorized as complete never-smoker predicted the presence of the *EGFR* mutation and current and former smoker predicted the presence of *KRAS* mutation. The SUV_max_ of FDG uptake in lung lesion were also significant parameters, while those of MTV and TLG were not significant. Of several parameters regarding tumor heterogeneity, 1/COV was the only significant factor which was not dependent on metabolic tumor diameter. The multivariate analysis showed never-smoker smoking status was the only significant factor for *EGFR* mutation, and that current and former smoker status was the only significant factor for KRAS mutation in lung cancer.

*EGFR* mutations have been linked patients with adenocarcinoma, lack of prior smoking history, females, and Asians. Our results demonstrate that never-smoking (no prior smoking history) was the most significant predictive factor for presence of the *EGFR* mutation, which corroborates previously observed trends [26]. The frequency of *KRAS* mutation is not associated with age, gender and smoking history (regardless of pack years) [27]. Therefore, *KRAS* mutation defines a distinct molecular subset of the disease. *KRAS* mutations were found in tumors from both former/current smokers and never smokers. They are rare in never smokers and are less common in East Asian Vs. US/European patients [27].

Our interest was how tumor metabolism (inferred from PET imaging) could add significant value to predict gene mutations. The most popular metabolic parameter in lung cancer is the SUV_max_, but SUV_max_ represents just a single point within the tumor even it is easy to measure. Several papers have reported relationship between FDG uptake in lung cancer and *EGFR* [17, 28–31] and *KRAS* gene mutations [17, 30]. Our study appears to show a positive correlation for SUV_max_ as significant factor for predicating *EGFR* mutation. We did not find a significant correlation between SUVmax to *KRAS*.

TLG and MTV were not significant factors for predicting gene mutations (Table 4). This suggests that gene mutation can occur regardless of the size or volume of lung lesion since TLG and MTV are proportional to tumor size. Therefore conventional assessment based on tumor size appeared to be limited for the prediction of gene mutation in lung cancer. One of the novelties of our study was to evaluate the relationship between tumor heterogeneity and gene mutation (Table 4). In regards to tumor heterogeneity, prior studies commonly include both partial volume effects and noise as heterogeneity [32]. Several FDG-PET/CT metrics regarding tumor heterogeneity correlated to *EGFR* mutation, but 1/COV appears to be the most reliable due to the lack of dependence on lesion size.

Yip et al. investigated the association between FDG-PET based radiomic features and somatic mutations in NSCLC. A significant relationship could be seen in SUVmax, MTV, minimum of SUV and several indexes obtained from texture based analysis for predicting EGFR mutations; on the other hand no index could be seen as predicting of KRAS mutations [33].

The key trend of representing tumor heterogeneity is texture based analysis [34, 35]. Several indexes were shown to have significant relationship with predicting EGFR gene mutations [33]. We did not adapt these methods in this study, because the methodology has not been standardized in terms of the software and indexes as described in each article. The 1/COV appears to have limited reliability in their robustness and repeatability, however we adopted PET edge method for tracing the edge of FDG uptake in tumor in order to minimize the measurement variance by the observer [36, 37].

The advantage for prediction of gene mutation in lung cancer was to select suitable therapeutic strategy for the patient with lung cancer, and it will be desirable if it could be possible by less invasive method. Stiles B et al. suggested that clinical stage IA lung cancer is frequently under staged in patients [38]. Goldstraw et al. reported that 30% to 70% of patients with completely resected disease experienced relapse and/or distant metastases [39]. It appeared that micrometastatic disease had already occurred at the some of cases with early-stage NSCLC.

Therefore, adjuvant and/or neoadjuvant cisplatin-based chemotherapy is advised for patients with early-stage disease [40–42]. Neoadjuvant therapy has advantages for downstaging the tumor before surgery and thus increasing the chances of a complete resection.

Several randomized clinical trials assessed the advantage of neoadjuvant treatment in patients with early stage disease. Although available data suggest a trend in survival benefit in preoperative chemotherapy, the majority of studies showed no statistically significant differences [43].

A phase II study of preoperative gefitinib in clinical stage I non-small-cell lung cancer demonstrated that tumor shrinkage was frequently seen in women, neversmokers and the EGFR expression (proven by biopsy) was a strong predictor of response [44]. Neoadjuvant chemotherapy has also been explored in patients with early-stage NSCLC. It is based on the rationale to be able to decrease micrometastases at distant sites and tumor burden preoperatively to increase resectability and overall survival.

In a systematic review, Nair et al. concluded that increased tumor FDG uptake is associated with poorer survival in patients with stage I NSCLC. FDG uptake has the potential to be used as a biomarker for identifying stage I patients who are at increased risk of death or recurrence and therefore could identify candidates for participation in future trials of adjuvant therapy [45].

However, one of the limitations of neoadjuvant therapy is the inability to confirm gene mutations prior to surgical resection. The prediction of gene mutation in lung cancer can be advantageous for selecting patients who would best benefit from neoadjuvant therapy.

The limitation of this study was that we have not yet obtained the result of patient's prognosis, therefore we could not report how FDG PET/CT could predict the prognosis nor its prognostic value when used in conjunction with, or compared against, smoking status.

## MATERIALS AND METHODS

### Study population

The Institutional Review Board and the Stanford Cancer Institute Scientific Review Committee approved this project and protocol. Written informed consent was obtained from all patients before participation in the study. Inclusion criteria were: 1) greater than 18 years-old at the time of radiotracer administration and 2) suspicion of lung cancer on preoperative CT scans by a board-certified radiologist specializing in thoracic imaging. Exclusion criteria were: 1) pneumonic form and central obstructive lesions on preoperative CT which was confirmed by a board-certified radiologist specializing in thoracic imaging.

### Clinical data collection

We collected the following clinical variables from each patient: age, histology, sex and smoking status. After review of the histology of NSCLC, we eliminated subtypes of adenocarcinoma including bronchioloalveolar carcinoma (BAC) as defined in the previous pathological classification for lung adenocarcinoma. Smoking status was categorized as never-smoker, former smoker or current smoker.

### EGFR and KRAS mutation testing

The tumor tissues were surgically resected for all patients. Mutation testing was done for both *EGFR* and *KRAS* using multiplex PCR followed by single nucleotide mutation detection using SNaPshot technology based on dideoxy single-base extension of oligonucleotide primers [46]. *EGFR* exons 18, 19, 20 and 21 were tested and *KRAS* exon 2. Mutations were combined irrespective of their location in the tested exons. Patients were categorized according to the mutation testing as *EGFR* mutated (*EGFR+*) and wild-type *EGFR*, and *KRAS*-mutated (*KRAS+*) and wild-type *KRAS*.

### PET/CT protocol

FDG-PET/CT scans were acquired by using a standard clinical protocol at two sites, Stanford University Hospital (SUH) and Veterans Administration Palo Alto Health Care System (VAPAHCS). PET/CT images were acquired using either GE Discovery LS PET/CT (slice thickness, 3–5 mm) (GE Healthcare, Waukesha, WI, USA) at Stanford or GE Discovery VCT (slice thickness, 3.75 mm) (GE Health care, Waukesha, WI, USA) at VAPAHCS. At both sites, patients fasted for a minimum of 6 hours, a dose of 12 to 17 millicuries (mCi) of FDG was administered, and patients were scanned from the skull base to mid-thigh using multiple bed positions every 5 minutes approximately 45 to 60 minutes after injection. CT-attenuated data were reconstructed using ordered subset expectation maximization for both scanner sites.

### Image analysis

**Figure 1:**
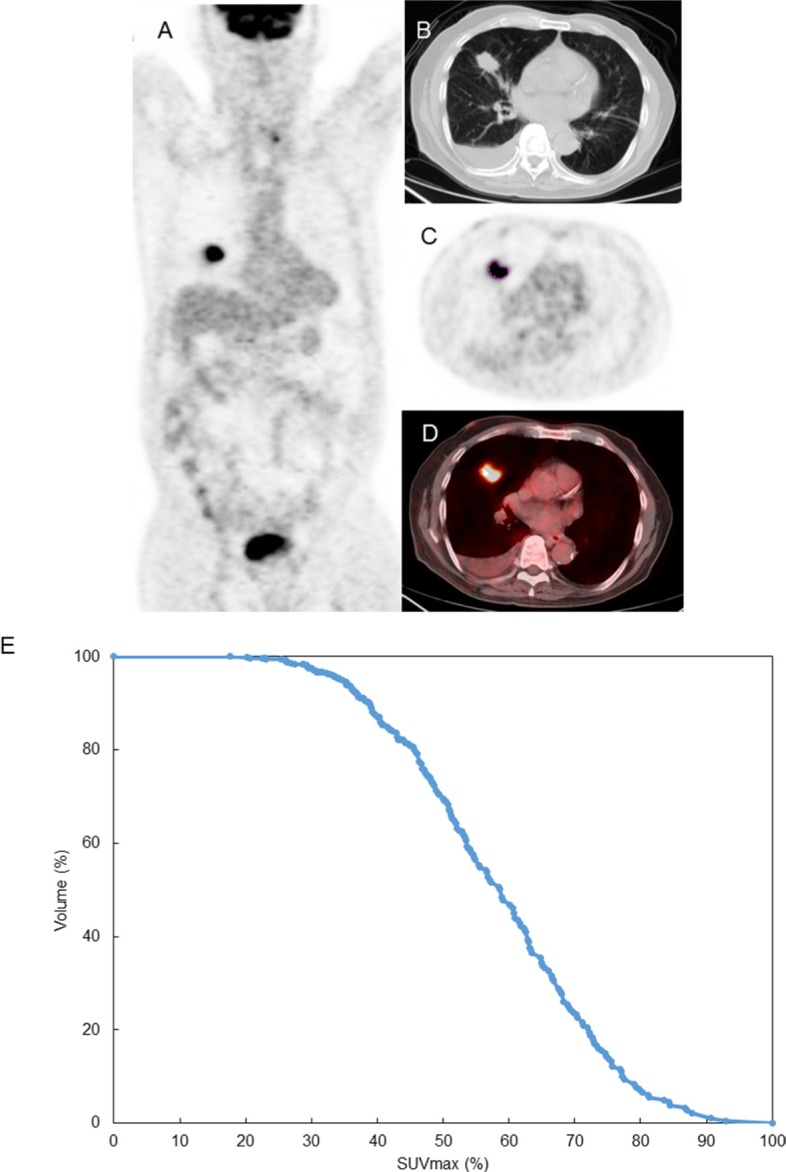
Images and measurement result of FDG PET parameter of lung tumor at right middle lobe (**A**) Sagittal whole body PET image, (**B**) CT portion of PET/CT, (**C**) PET image (plotted the edge of lung tumor), and (**D**) fused PET and CT image, (**E**) cumulative SUV-volume histograms (CSH) : The area under the curve (AUC) of this plot (AUC-CSH) was 0.58.

Representative images are shown in Figure 1. Images were reviewed by two board-certified Nuclear Medicine physicians (RM, AQ) with 8 and 15 years experience respectively. MIMvista 6.2 software (MIMvista Corp, Cleveland, OH, USA) was used to select and measure structures throughout the body using the region-of-interest (ROI) tool within the software. Circular ROIs with a diameter of 10mm were drawn on transaxial FDG-PET/CT images using the fusion CT scan as an anatomical guide. Background FDG uptake measurement with 10mm ROI was conducted for the upper, middle and lower field of lung in both lungs (if a lung lesion happened to exist in the nearby lung field, the measurement was not performed due to the possibility of affected by tumor FDG uptake). For the aortic blood pool, a circular ROI with 10mm of diameter was placed centrally within the ascending aorta. For SUV measurements of malignant lesions on PET images, CT images of these lesions were used to confirm the exact location of suspected malignant lesions, with reference to diagnostic chest CT. The PETedge tool within MIMvista 6.2 was used with manual adjustment where needed by consensus of two nuclear medicine physicians for measurements of lung tumor. The longest diameter of identified FDG uptake area by the PETedge tool was measured and was regarded as the metabolic tumor diameter rather than the true diameter. The maximum SUV (SUV_max_), average SUV (SUV_mean_), standard deviation of SUV metabolic tumor volume (MTV) and total lesion glycolysis (TLG: product of MTV and SUV_mean_) within a volume of interest (VOI) were recorded. By using these measurement results, inverse coefficient of variation [1/COV, calculated as (SUV_mean_/SD) ×100%] were additionally calculated as a marker of tumor heterogeneity. Additional metrics for tumor heterogeneity included the calculation of cumulative SUV-volume histograms (CSH) obtained by plotting the percent (SD), volume of a tumor with an SUV above a certain threshold against that threshold, which is varied from 0 to 100% of SUV_max_. The area under the curve (AUC) of this plot (AUC-CSH) is a quantitative index of uptake heterogeneity, where lower values correspond with increased heterogeneity [32].

Tumor heterogeneity analyses were performed only on cases with a metabolic tumor diameter of 10mm or more (117 of 131 cases) in order to have an adequate number of pixels within a region- or volume-of-interest.

### Statistical analysis

Mann-Whitney's test was used to compare the difference of PET uptake in normal lung, and PET parameters according to the gene mutation result. We used univariate analysis and multivariate analysis to investigate the relationship between the parameters regarding FDG uptake for lung lesions and the presence of *EGFR* and *KRAS* mutations. We also used univariate analysis for the relationship between several indexes and FDG uptake in normal lungs. All statistical analyses were done with Stata 11 (Stata, College Station, TX). Calculated *p*-values were two-sided with a p <.05 considered statistically significant.
